# Seasonal Variability of *Juniperus communis* L. Berry Ethanol Extracts: 1. In Vitro Hydroxyl Radical Scavenging Activity

**DOI:** 10.3390/molecules25184114

**Published:** 2020-09-09

**Authors:** Jozef Fejér, Ivan Kron, Daniela Gruľová, Adriana Eliašová

**Affiliations:** 1 Faculty of Humanities and Natural Sciences, Department of Ecology, University of Prešov, 17. Novembra 1, 08001 Prešov, Slovakia; jozef.fejer@unipo.sk (J.F.); adriana.eliasova@unipo.sk (A.E.); 2 JNT Ltd. SK 04001 Košice, Slovakia; kron.ivan@gmail.com

**Keywords:** ethanol extracts, antioxidant activity, hydroxyl radical, terpene hydrocarbons, total phenols

## Abstract

In the present study, in vitro hydroxyl radical scavenging activities, total phenols and terpene contents in 70% ethanol extracts were evaluated. Samples of crushed (CB) and non-crushed ripe juniper berries (NCB) collected at five localities in North-East Slovakia during the years 2012–2014 were compared. Standard or modified methods for determining phytochemical and antioxidant activity were used together with a novel method for the evaluation of the results after the correction of the measured values per gram of dry matter (DM). Statistically significantly higher DM contents (average values for three years) were found in the CB extracts (ranging from 18.86 to 21.91 mg/mL) in comparison to those for NCB (ranging from 2.59 to 9.90 mg/mL). Depending on the localities and years, the hydroxyl radical scavenging activity ranged from 15.52 to 32.85% for NCB and from 65.59 to 88.12% for CB, respectively. The contents of total phenols ranged from 43.75 to 246.75 mg gallic acid equivalents (GAE)/L (NCB) and from 151.84 to 278.19 mg GAE/L (CB), respectively. However, the higher content of total phenols per gram of DM was found in the NCB extracts (8.49–42.23 mg GAE/g DM) and then in CB (6.87–18.77 GAE/g DM). The results obtained in this study showed a higher efficiency for extraction from juniper berries in 70% ethanol if the pericarp was disrupted in comparison to that achieved with the maceration of intact berries.

## 1. Introduction

Antioxidants include free radical scavengers, which quench singlet oxygen, activators of peroxides and other reactive oxygen species (ROS); metal ion chelators; quenchers of secondary oxidation products; and inhibitors of pro-oxidative enzymes, among others [1]. Free radicals are formed during normal cell metabolism [2]. They are derived from oxygen and other elements as well [3]. Reactive oxygen species, such as superoxide radicals (O_2_•-), hydrogen peroxide (H_2_O_2_), hydroxyl radicals (HO•), and singlet oxygen (^1^O_2_), are generated as byproducts of normal metabolism [4]. The hydroxyl radical is the neutral form of the hydroxide ion. It is the most reactive oxygen radical known [5]. The hydroxyl radical reacts with most biomolecules and causes serious damage [3]. It is short-lived but reacts very rapidly with no selectivity and reacts with almost every type of molecule found in living cells, including sugars, amino acids, phospholipids, DNA, organic acids and fatty acids [6]. To detect the ability to create hydroxyl radicals, several methods can be employed. One of them is the deoxyribose test. This method includes a mixture of ferric chloride (FeCl_3_) and ethylenediamine tetra acetic acid (EDTA), which, in the presence of ascorbic acid, forms Fe^2+^-EDTA and an oxidized form of ascorbic acid. After the addition of hydrogen peroxide (H_2_O_2_), Fe^3+^-EDTA and HO• are formed. This is the so-called Fenton reaction, which generates the highly reactive hydroxyl radical (Fe^2+^ + H_2_O_2_ → Fe^3+^ + OH^−^ + HO•). Hydroxyl radicals that are not scavenged by any component of the mixture attack the deoxyribose and degrade it into several fragments. Some of these fragments are capable of reacting with thiobarbituric acid after heating and in an acidic pH, originating a pink pigment that can be quantified by spectrophotometry [7].

Various endogenous antioxidant defense mechanisms play an important role in the elimination of ROS and lipid peroxides and, therefore, protect the cells against the toxic effects of ROS and lipid peroxides [8].

*Juniperus communis* is an evergreen, perennial, long-lived coniferous plant having the largest distribution of any woody plant in the cool temperate geographical regions. It is spread globally and exhibits a wide range of ecological adaptations. A wide geographical distribution is the main reason for the remarkable variation in the morphological characteristics and chemical composition of the secondary metabolites [9,10].

Juniper leaves and berries were traditionally used in folk medicine [11], also likely due to their antioxidant properties. Antioxidants found in foods protect cells from damage caused by free radicals by various mechanisms [1]. The in vitro antioxidant activity of water and ethanol extracts of the *J. communis* fruit was investigated and compared with that of commercially available antioxidants. Both juniper extracts exhibited strong total antioxidant activity [12].

In the present study, we focused on the evaluation of the efficiency of a 70% ethanol extract of non-crushed (NCB) and crushed juniper ripe berries (CB) obtained from natural populations of North-East Slovakia within three years, the antioxidant activity of ethanol extracts against the hydroxyl radical, and the determination of the basic constituents of the extracts for which antioxidant activity is predicted (the quantities of total phenols and terpene hydrocarbons in the extracts). Due to the different dry matter contents and for better comparison of our results, the antioxidant activity values, total phenol values and terpene values were recalculated per gram of dry matter.

## 2. Results

### 2.1. The Influence of Extraction Method on Total Amount of Extracted Substances

The method of berry preparation before extraction influenced the total amount of extracted substances in the ethanol extracts. As shown in Table 1 and Table 2, crushed fruit extracts have a higher dry matter content on average for three years, ranging from 18.86 (±4.24) to 21.91 (±1.19) g/L. In each of the monitored years, the dry matter content varied from 13.91 g/L (locality Hôrka, year 2012) to 23.84 g/L (locality Lačnov, year 2012). In the NCB extracts (macerates), the average amounts of solids varied from 2.59 (±0.94) to 9.90 g/L (±5.77). Differences in the dry matter (DM—see Paragraph 4.1. in Methods) contents in the NCB extracts were also found between localities and years. The DM content ranged from 1.39 g/L (locality Hôrka, year 2014) to 16.55 g/L (locality Kamienka, year 2012). This high variability is probably related to the thickness of the juniper berry peel, which apparently affected the amount of substances extracted. Statistically significantly higher amounts of DM were present in the CB extracts than in the macerates (*p* < 0.001). No statistical differences in DM between localities and years were found for both types of extracts.

### 2.2. Hydroxyl Radical Scavenging Activity

The antioxidant effects against hydroxyl radicals ranged from 15.52% (locality Kamienka, year 2013) to 32.85% (locality Lačnov, year 2012) for the NCB extracts (Table 1). During the three evaluated years, the average antioxidant activity was in the range of 21.26% (±2.55) to 31.30% (±2.82) (Table 2). The antioxidant activity of the CB extracts was higher and also varied depending on the locality and year of harvest within the range of 65.59% (locality Zbojné, year 2013) to 88.12% (locality Lačnov, year 2014) (Table 1). During the three evaluated years, the average antioxidant activity was in the range of 75.74% (±9.10) to 82.82% (±1.92) (Table 2).

The juniper extracts from 2014 showed statistically significantly higher antioxidant activity in comparison to those from 2012 (*p* = 0.013). Between the years 2012 and 2013, as well as between the years 2014 and 2013, the differences were not statistically significant. At the values recalculated to dry matter, no statistically significant differences in antioxidant activity were found between the extracts from different localities (*p* = 0.525) and the years (*p* = 0.257) (See Table 2). On contrary, the crushed fruit extracts from the year 2014 showed statistically significantly higher activity compared to the extracts from the years 2012 and 2013 (*p* = 0.038). The difference between the 2012 and 2013 extracts was not significant. There were also found no statistically significant differences between the CB extracts from different localities (*p* = 0.446) and the years (*p* = 0.949) at the values recalculated to dry matter. The CB extracts showed statistically significantly higher antioxidant activity against the hydroxyl radical in comparison to the NCB extracts (*p* < 0.001) (see Table 2).

Gallic acid was included as the standard, and its antioxidant activity was determined in both series (NCB and CB extracts). In a series of experiments with the NCB extracts, it showed antioxidant activity of 41.24% (±3.91). In a series of experiments with the CB extracts, its activity against the hydroxyl radical was found to be 43.60% (±5.98), which shows good agreement between both determinations. There was a statistically significantly lower antioxidant activity in the NCB extracts from all localities in comparison to that of gallic acid. Differences were also found in the relationship with the origins of the berries (*p* < 0.001), (Table 3).

The CB extracts showed statistically significantly higher activity against hydroxyl radicals in comparison to standard gallic acid. Statistically significant differences were also found between the CB extracts from individual localities (*p* < 0.001) (Table 4).

### 2.3. Content of Total Phenols

The contents of total phenolic substances of the NCB extracts from the studied localities varied from 43.75 mg gallic acid equivalents (GAE)/L (locality Miľpoš, year 2014) to 246.75 mg GAE/L (locality Kamienka, year 2012). The values recalculated to dry matter varied from 8.49 (Hôrka locality, year 2012) to 42.23 mg GAE/g DM (Hôrka locality, year 2014) (Table 1). During the three evaluated years, the average values of the total phenolic contents were in the range of 48.87 (±5.94) to 154.13 mg GAE/L (±85.89), or 15.85 (±4.36) to 23.67 mg GAE/g DM (±17.06), respectively (Table 2). In the CB extracts, the amount of total phenols was higher than in the NCB extracts and ranged from 151.84 (Hôrka locality, year 2013) to 278.19 mg GAE/L (locality Lačnov, year 2012), and the values recalculated to dry matter ranged from 6.87 (Kamienka locality, year 2014) to 18.71 mg GAE/g DM (Hôrka locality, year 2012) (Table 1). During the three evaluated years, the total phenol average was found to be in the range of 201.30 (±35.35) to 251.21 mg GAE/L (±34.73), or 9.30 (±1.67) to 12.19 mg GAE/g DM (±5.97), respectively (Table 2). The statistical evaluation of the obtained and non-recalculated data (in mg GAE/L) by analysis of variance showed statistically significantly higher contents of total phenols in the NCB extracts from locality Kamienka, in comparison to those in the extracts from localities Zbojné and Miľpoš (*p* = 0.086). Between the studied years, the differences were not significant (*p* = 0.413). The amounts of total phenolic compounds in the NCB extracts (recalculated data in mg GAE/g DM) did not reveal any statistically significant differences among individual localities (*p* = 0.331). The contents of total phenols recalculated to dry matter were significantly higher in the year 2014, compared to in the year 2013 (*p* = 0.016). There was not a significant difference between the year 2012 and the years 2013 and 2014. In the CB extracts, statistically significantly higher contents of phenolic compounds (in mg GAE/L) were found in the locality Lačnov (*p* = 0.014). Highly evident differences were found between all the years under review (*p* ˂ 0.001), with the highest amount recorded in the year 2012 and lowest in the year 2013. For the recalculated values (in mg GAE/g DM) were found statistically significant differences in the locality Hôrka compared to Kamienka and Zbojné, and between localities Lačnov and Zbojné (*p* = 0.086). The contents of total phenols recalculated to dry matter were significantly higher in the year 2012, compared to those in the years 2013 and 2014 (*p* = 0.005). There was not a significant difference between the years 2013 and 2014. For comparison, in our experiments, we used a sample of *Juniperus oxycedrus* (a commercial sample from the Prelika distillery, Prešov, Slovakia, with the berries originating from Albania). Its total phenol contents were 78.20 (±6.93) (non-crushed berries) and 19.48 (±7.04) mg GAE/L (crushed berries), and after recalculation to DM, they were 11.76 (±1.04) and 1.01 (±0.30) mg/g DM, respectively, lower than in *J. communis* (Table 1). In spite of that, the antioxidant activity was comparable to that of *J. communis*. The *J. oxycedrus* NCB extracts showed 31.12% (±2.96) and CB extracts 81.27% (±5.44) antioxidant activity, respectively. Recalculation by dry matter gives antioxidant activity of 4.68 (±0.45) %/g DM (NCB extracts) and 4.20 (±0.28) %/g DM (CB extracts), respectively.

### 2.4. Content of Terpene Hydrocarbons in Extracts

**Figure 1:**
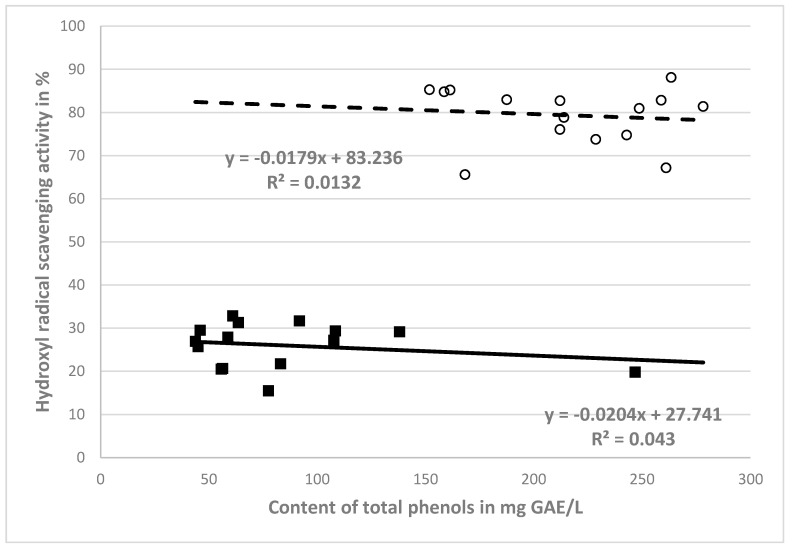
Correlations between antioxidant activity and phenol content of NCB (■) extracts and CB (○) extracts.

The higher non-corrected antioxidant activity of the CB extracts (Table 1 and Table 2, Figure 1) forced us to look for constituents besides phenols possibly contributing to the antioxidant activity. Therefore, terpene hydrocarbon contents were determined only in the CB extracts due to expected lower values in the NCB extracts. The contents of terpene hydrocarbons expressed as milligrams of sabinene equivalents per gram of the extract DM were determined. Based on GC-FID analysis, α-pinene was the most abundant among the monoterpene and sesquiterpene-type components of the extracts. Humulene, β-caryophyllene, terpinen-4-ol, sabinene and β-myrcene were present in relatively large amounts as well [10]. The total terpene hydrocarbon content in the extracts ranged from 38.68 (locality Miľpoš, year 2012) to 79.33 mg/g DM (locality Hôrka, year 2014) (Table 2). During the three evaluated years, the average content was found to range from 50.28 (±13.41) to 68.29 mg/g DM (±9.76) (Table 4). The differences in the contents of terpene hydrocarbons between extracts from the different localities and evaluated years were not statistically significant (*p* = 0.181 and *p* = 0.166, respectively).

### 2.5. Correlation Analysis

In order to assess the dependence of antioxidant activity (%) on the amount of total polyphenols (mg GAE/L) in the NCB and CB extracts, a correlation analysis was performed—see Figure 1. From Figure 1, it is clear that negative correlations (r = −0.207 for non-crushed berry extract and r = −0.115 for crushed berry extract) occurred for the relationships between the antioxidant activities against hydroxyl radicals and the phenolic contents of the crushed and non-crushed berry extracts.

**Figure 2:**
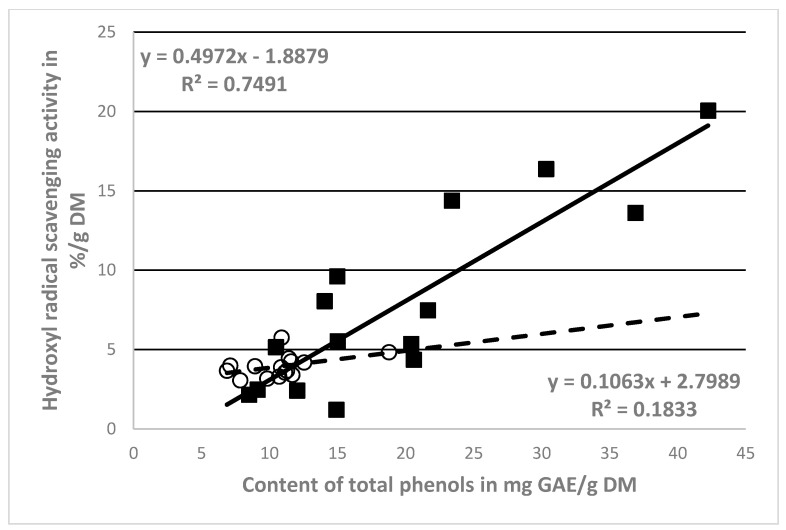
Correlations between antioxidant activity and phenol content corrected by DM of NCB (■) extracts and CB (○) extracts.

However, recalculated values of the antioxidant activity (%/g DM) and the total phenolic substance contents (mg/g DM) per gram of DM were used for more objective assessment (Table 1 and Table 2). Indeed, a high positive correlation between the hydroxyl radical scavenging activity (%/g DM) and phenol content in the ethanol extracts of NCB (mg/g DM) was found (r = 0.866), contrarily to what was found for the CB extracts (r = 0.428)—see Figure 2.

**Figure 3:**
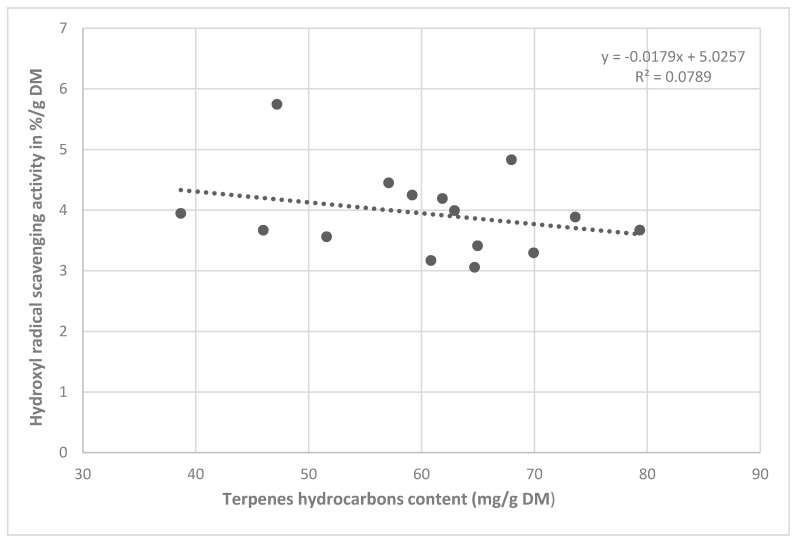
Correlation between antioxidant activity and terpene content in CB extracts.

The content of terpene hydrocarbons was only analyzed for the CB extract. On the contrary, the correlation analysis confirmed an indirect proportional participation of the essential oil content in the antioxidant activity (r = −0.281, Figure 3).

## 3. Discussion

Many substances (usually secondary metabolites of plants, such as phenols, terpenes, etc.) exert their inhibitory effects against oxidation processes through different mechanisms and with varied activities. They are broadly classified by their mechanisms of action as primary and secondary antioxidants. Primary antioxidants such as tocopherols and some phenolic compounds inhibit the chain reaction of oxidation by acting as hydrogen donors or free radical acceptors and the generation of more stable radicals. Secondary antioxidants prevent or retard oxidation by suppressing oxidation promoters, including metal ions, singlet oxygen, pro-oxidative enzymes and other oxidants [13,14].

The content of total polyphenols in plants varies considerably. Researchers evaluated the amounts of total phenolic compounds in 233 plant species [15]. They detected contents within the range of 0.19 to 101.33 mg GAE/g dry matter. In other study [16] of 92 plant extracts, the amount of total phenolics varied widely and ranged from 0.2 to 155.3 mg GAE/g DM. The berries contained relatively higher amounts of phenolics than cereals or vegetables. In the berries from *Juniperus sibirica*, the content of phenolic compounds was found to be 62.13 (±9.47) mg GAE/g [17]. Lower values were reported for total phenols in *Juniperus communis* extracts, ranging from 6.86 (±0.11) [18] to 18.5 (±0.62) mg GAE/g [19].

In the evaluated NCB juniper extracts, the total phenolic compounds varied from 43.75 to 246.75 mg GAE/L (recalculated by dry matter, from 8.49 to 42.23 mg GAE/g DM). The CB extracts contained from 151.84 to 278.19 mg GAE/L (calculated by dry matter, from 6.87 to 18.77 mg GAE/g DM). Our data are quite close to the literature sources, but differences appeared in the use of crushed and non-crushed berries for extraction with 70% ethanol. The origin of the juniper berries and the evaluated year must be considered.

The ethanol CB extracts contained significantly more total phenolic compounds than the NCB extracts. The differences in the phenol contents of the NCB extracts between localities were not conclusive. In the case of the CB extracts, there was a significant increase in total phenols in the berry extract obtained from one locality (Lačnov) compared to those of the extracts from the other sites. In both types of extracts, the phenol content was proven to vary significantly between the years evaluated.

Researchers [20] evaluated the antioxidant activities against hydroxyl radicals in fruit juices from different cultivars of blackberries, blueberries, cranberries, raspberries and strawberries. In the juices, the antioxidant capacity values against hydroxyl radicals ranged from 58.7% (blueberries) to 72.0% (blackberries). They found differences between the varieties of the evaluated fruit species. In a different study [17] was found a relatively high IC50 value of 479.53 (±18.60) µg/mL for the hydroxyl scavenging activity of a juniper needle extract. However, 50% inhibition was not achieved with a juniper berry extract. The antioxidant activity against hydroxyl radicals of various culinary herbs and spices was studied and examined by the deoxyribose degradation assay, expressed as mannitol equivalents [19]. They ranked the herbs from the best to worst in the order basil > laurel > parsley = juniper = fennel > anise seed > cardamom = ginger = cumin. Juniper extracts were approximately in the middle.

The values corrected by the dry matter content showed that the extraction of compounds only from the peel and part of the berry provided more compounds active against hydroxyl radicals (Table 1) than the hydroxyl radical antioxidant activity observed in our study; depending on the type of extract (NCB or CB) and origin of the juniper berries, it ranged from 15.52% to 32.85% (recalculated by dry matter, from 1.20 to 20.05%/g DM) for the NCB extracts and from 65.59% to 88.12% (recalculated by dry matter, from 3.06 to 5.75%/g DM) for the CB extracts, respectively. The values for the CB extracts are in very good agreement with [20], determining activity in fruit juices. The antioxidant activity was significantly higher in the CB extracts than in the NCB extracts due to the higher contents of dry matter containing more active compounds. However, the values for the extracts from the crushed berries had to be corrected. The activity of the NCB extracts was significantly lower in comparison to that of the gallic acid standard, while the CB extracts showed significantly higher activity. There were also significant differences in activity against the hydroxyl radical depending on the locality from which the berries were collected, as well as between the evaluated years of harvesting.

The correlation analysis showed a highly evident effect of phenolic compounds on the antioxidant activity of the NCB extracts (r = 0.865, Figure 2). The CB extracts were found to have a lower correlation (r = 0.429, Figure 2). Consequently, other components of the extracts have been implicated in the antioxidant activity. NCB extracts contain only the substances from the skin and parts of the insides of the berries (phenols, including dyes). This indicates a higher relative antioxidant activity (%/g DM). Other substances, including polysaccharides, also entered the extract from the crushed fruit, which is also shown by the significantly higher dry matter content (Table 1).

In the literature, a number of investigations dealing with the in vitro antioxidant activity of monoterpenes, diterpenes or essential oils are reported. The results describe some terpenes as very effective antioxidants, e.g., γ-terpinene [21]. The ability of the essential oils of *Thymus marschallianus* and *Thymus proximus* to scavenge hydroxyl radicals was reported [22]. The activity of both essential oils was dose dependent, and they mainly constituted thymol, p-cymene and γ-terpinene. In a different study [23], the HO• free radical scavenging activity of the essential oil and its compounds from berry samples of the *J. communis* subsp. *Hemisphaerica* was measured with the procedure described by [24]. They determined the deoxyribose assay antioxidant activity of the essential oil to range from 0.77% (±0.86) to 7.10% (±1.15)—depending on the concentration (from 0.05 to 1.00 μL/mL) used. For selected essential oil components, depending on the concentration used, it was found that the antioxidant activity of β-pinene was 25.23–47.60%, that of limonene was 12.60–40.60% and that of γ-terpinene was 6.97–17.07%. There was no activity for sabinene at the lowest concentration (0.05 µL/mL) and only 1.27% at the highest concentration (1.00 µL/mL). Sabinene at a concentration of 0.2 µL/mL exhibited 17.0% antioxidant activity. The α-pinene exhibited an antioxidant effect of 1.20% activity for a concentration as low as 0.2 µL/mL and only 4.10% activity at the highest concentration.

A similar effect of the antioxidant activity in the deoxyribose assay in the study of essential oil components (β-pinene, limonene, γ-terpinene, sabinene and α-pinene) of *Juniperus excelsa* subsp. *excelsa* and *J. excelsa* subsp. *polycarpos* was reported by [25]. The essential oil of *J. excelsa* subsp. *Polycarpos*, in which there was a high content of α-pinene (78.26%) and very low or trace amounts of β-pinene, limonene, γ-terpinene and sabinene, did not show antioxidant activity in the deoxyribose assay. On the other hand, the essential oil from small leaves of this species, in which the above-mentioned components were detected, had an antioxidant activity of 3.9–35.5%. The essential oil of *J. communis* subsp. *Hemisphaerica*, which exhibited antioxidant activity, contained limonene, γ-terpinene and sabinene [23]. It follows that some terpenes found in the oil exhibit antioxidant activity against HO•.

In our study, the total essential oil content recalculated for sabinene was determined in *J. communis* crushed juniper berry ethanol extracts, whose antioxidant activity against the hydroxyl radical negatively correlated with it (r = −0.281, Figure 3). From the obtained results, we can assume an interaction between several components of the essential oil and their influence on the resulting activity against the hydroxyl radical in the case of the non-crushed fruit extracts. Therefore, we cannot exclude a certain proportion of some of the essential oil components described in the literature for this activity. In a previous study were detected the components β-pinene, limonene and sabinene in the essential oil of berry samples from the studied localities, which could be involved in the anti/pro-oxidant effects [10]. The fact that the antioxidant activity of the *J. oxycedrus* extracts (31% for non-crushed berries and 81.27% for crushed berries) was comparable to the activity of the *J. communis* extracts, despite the lower contents of total phenolic compounds in *J. oxycedrus* (11.76 mg/g DM in non-crushed berries and 1.01 mg/g DM in crushed berries), in comparison to those in *J. communis* (19.63 mg/g DM in non-crushed berries and 10.76 mg/g DM in crushed berries). An important point when assessing the antioxidant activity of plant antioxidants is to consider their interaction with other antioxidants [21]. From the obtained results, we can assume an interaction between several components of the essential oil and their influence on the resulting activity against the hydroxyl radical in the case of the non-crushed fruit extracts.

For an objective assessment of the dependence of the antioxidant activity on phenols and essential oil components, the values found were recalculated per gram of dry matter. The correlation analysis confirmed the significant antioxidant effect of the phenolic compounds in the NCB extracts. A lower correlation coefficient was found for the CB extracts. Consequently, other components extracted into ethanol could also contribute to the antioxidant activity. In the literature, the effect of terpenes on antioxidant activity is reported, and therefore, the analysis of the components in essential oil recalculated for the sabinene contained in the crushed fruit extracts was performed. However, the correlation analysis showed a negative correlation (r = −0.281, Figure 3). It is questionable whether some of the terpenes contained in the extracts contributed to the antioxidant or pro-oxidant activity against the hydroxyl radical.

## 4. Materials and Methods

### 4.1. Plant Material and Extract Preparation

The ripe juniper berries were collected in five localities (Hôrka, Kamienka, Miľpoš, Lačnov and Zbojné) in the northeast region of the Slovak republic during the last ten days of September within the three years 2012–2014 and naturally dried, as was described previously [10].

Five grams of dried crushed berries (CB) ground with a pestle in a porcelain mortar or non-crushed berries (NCB) were mixed with 100 mL of 70% ethanol. The extractions were carried out with occasional stirring for 72 h at room temperature. The obtained extracts were filtered over a KA 1-M filter (very fast, papírny pernštejn, CZ). The dry matter (DM) content was determined in the filtrates after drying the extract in Petri dishes in an oven at 105 °C for three hours, with each extraction performed in triplicate.

All the used chemicals were of the highest quality: 70% ethanol (Centralchem, chemical trading company, Slovakia). Double distilled water (DDW) was used for the preparation of solutions. The absorbances of the solutions for the various test assays were determined in 1 cm quartz cells with a spectrophotometer, Shimadzu type UV-1800 (manufacture, Shimadzu, Japan).

### 4.2. Hydroxyl Radical Scavenging Activity

The deoxyribose assay was applied to measure HO• scavenging capacity [26] with small modifications [27]. The antioxidant activity of each sample, expressed as percentage of inhibition (POI), was calculated. All determinations of antioxidant activity against hydroxyl radicals in the samples were performed at least four times.

### 4.3. Total Phenolics

The total phenolic contents of the ethanol extracts of leaves were determined with the Folin–Ciocalteu reagent (FCR, Merck) according to [28] with slight modifications [27]. The amounts of polyphenols in the samples were calculated as gallic acid equivalents (GAE). All determinations of total polyphenols in samples were performed at least four times.

### 4.4. Content of Terpene Hydrocarbons in Extracts

The determination of the terpene hydrocarbon contents in the CB extracts was performed using a gas chromatographic system, Carlo Erba GC 6000 Vega Series 2, equipped with an ICU-600 program controller, an EL-580 flame ionization detector (FID), a Spectra Physics SP 4270 integrator, and a SolGel-WAX GC Capillary Column (60 m × 0.25 mm i.d.; film thickness, 0.25µm). An injector was heated to 200 °C, and a flame ionization detector was heated to 300 °C. The column temperature was maintained at 50 °C for 5 min and then programmed to increase to 200 °C at a rate of 5 °C/min and held at this temperature for 10 min; the injection volume was 1 µL; the split ratio was 1:20; nitrogen was used as a carrier gas (1.5 mL/min). The identification of monoterpene and sesquiterpene hydrocarbons was performed on the basis of the co-injection of some commercially available standards. Quantification was conducted with the peak area values obtained from GC-FID, using sabinene as an external standard. The content of terpene hydrocarbons was expressed as milligrams of sabinene equivalents per gram of the extract dry matter.

### 4.5. Statistical Analysis

The statistical software Statgraphics 5.0 and multifactorial analysis of variance (ANOVA) with the LSD (Least Significant Difference) 95% method were used for statistical analysis. The analysis of variance was conducted on the samples to determine variations in hydroxyl radical scavenging activity between the extracts, localities and years. The software Statistica 12 was used for correlation analysis (*p* ˂ 0.05) between the hydroxyl radical scavenging activity and content of total polyphenols and essential oil components in the extracts.

## 5. Conclusions

The results obtained in this study showed a higher efficiency of extraction from juniper berries in 70% ethanol if the pericarp was disrupted in comparison to that achieved with the maceration of intact berries. Thus, crushed berry extracts contained more dry matter than the extracts in which the macerated berries were intact. This could be crucial for the processing of the juniper fruits for different purposes. In general, differences in the dry matter contents in the NCB extracts were found between localities and years. This is probably related to the thickness of the juniper berry peel, which apparently affected the amount of substances extracted. The antioxidant activity of the CB extracts was higher as was the amount of total phenols in comparison to that of the NCB extracts. Differences were also found in the relationship with the origins of the berries and between the years of harvest. Our study will continue with the assessment of the antioxidant activity of juniper berry ethanol extracts against superoxide radicals and FRAP.

## Figures and Tables

**Table 1:** Antioxidant activity against hydroxyl radicals, dry matter, total phenols and contents of essential oil components in 70% ethanol extracts of juniper berries.

Parameter	Locality Zbojné			Locality Hôrka			Locality Miľpoš			Locality Kamienka			Locality Lačnov		
	Year			Year			Year			Year			Year		
	2012	2013	2014	2012	2013	2014	2012	2013	2014	2012	2013	2014	2012	2013	2014
**Non-crushed ripe juniper berries extracts**															
POI-Hydroxyl (%)	20.51	25.72	29.52	27.20	21.75	27.93	31.30	20.67	26.94	19.80	15.52	29.17	32.85	29.36	31.68
POI-Hydroxyl (%/g DM)	13.61	8.04	9.61	2.15	5.34	20.05	5.15	5.52	14.38	1.20	2.41	4.35	16.37	2.47	7.47
DM (g/L)	1.51	3.20	3.07	12.68	4.07	1.39	6.07	3.75	1.87	16.55	6.44	6.70	2.01	11.88	4.24
Phenols (mg GAE/L)	55.7	44.95	45.95	107.65	83.05	58.70	63.55	56.27	43.75	246.75	77.45	138.00	60.95	108.35	91.75
Phenols (mg GAE/g DM)	36.89	14.05	14.97	8.49	20.41	42.23	10.47	15.01	23.40	14.91	12.03	20.60	30.32	9.12	21.64
**Crushed ripe juniper berries extracts**															
POI-Hydroxyl (%)	82.83	65.59	82.95	67.17	85.29	74.77	73.76	85.19	78.85	80.95	82.72	84.78	81.38	76.05	88.12
POI-Hydroxyl (%/g DM)	3.56	3.06	3.95	4.83	3.99	3.30	3.17	5.75	4.25	3.67	4.45	3.67	3.41	3.89	4.19
DM (g/L)	23.26	21.45	21.02	13.91	21.37	22.69	23.27	14.83	18.47	22.07	18.59	23.09	23.84	19.57	21.02
Phenols (mg GAE/L)	258.81	168.25	187.50	261.05	151.84	242.85	228.63	161.38	213.89	248.62	212.12	158.61	278.19	212.03	263.40
Phenols (mg GAE/g DM)	11.13	7.84	8.92	18.77	7.11	10.70	9.82	10.88	11.58	11.27	11.41	6.87	11.67	10.84	12.53
Terpene hydrocarbon content (mg/g DM)	51.59	69.93	57.09	64.70	60.83	79.33	38.68	47.20	64.97	67.97	59.17	73.62	62.92	45.99	61.85

POI = percentage of inhibition; GAE = gallic acid equivalents; Hydroxyl % = data expressed as percent inhibition of OH• radical production in the presence of 10 µL of extract; Hydroxyl %/g DM and Phenols mg GAE/g DM = data calculated for the dry matter.

**Table 2:** Average values (3 years, 2012–2014) of antioxidant activity against hydroxyl radicals, dry matter, total phenols and contents of essential oil components in 70% ethanol extracts of juniper berries.

Parameter	Locality Zbojné		Locality Hôrka		Locality Miľpoš		Locality Kamienka		Locality Lačnov	
	Average	SD	Average	SD	Average	SD	Average	SD	Average	SD
	**Non-crushed ripe juniper berries extracts**									
POI-Hydroxyl (%)	25.25	±1.43	25.62	±3.60	26.30	±0.86	21.26	±2.55	31.30	±2.82
POI-Hydroxyl (%/g DM)	10.42	±1.61	9.18	±0.89	8.35	±0.98	2.64	±0.05	8.77	±1.86
DM (g/L)	2.59	±0.94	6.05	±5.90	3.90	±2.10	9.90	±5.77	6.04	±5.18
Phenols (mg GAE/L)	48.87	±5.94	83.13	±24.48	54.52	±10.01	154.13	±85.89	87.02	±19.28
Phenols (mg GAE/g DM)	21.99	±12.98	23.67	±17.06	16.28	±6.54	15.85	±4.36	20.38	±10.68
	**Crushed ripe juniper berries extracts**									
POI-Hydroxyl (%)	77.12	±10.00	75.74	±9.10	79.15	±5.74	82.82	±1.92	81.85	±6.05
POI-Hydroxyl (%/g DM)	3.52	±0.45	4.04	±0.77	4.39	±1.29	3.93	±0.45	3.83	±0.39
DM (g/L)	21.91	±1.19	19.32	±4.73	18.86	±4.24	21.25	±2.36	21.48	±2.17
Phenols (mg GAE/L)	204.85	±47.71	218.58	±58.51	201.30	±35.35	206.45	±45.27	251.21	±34.73
Phenols (mg GAE/g DM)	9.30	±1.67	12.19	±5.97	10.76	± 0.88	9.85	±2.58	11.68	±0.85
Terpene hydrocarbon content (mg/g DM)	59.54	±9.41	68.29	±9.76	50.28	±13.41	66.92	±7.28	56.92	±9.48

POI = percentage of inhibition; GAE = gallic acid equivalents; Hydroxyl % = data expressed as percent inhibition of OH• radical production in the presence of 10 µL of extract; Hydroxyl %/g DM and Phenols mg GAE/g DM = data calculated on the dry matter; SD = standard deviation.

**Table 3:** Multiple range analysis for antioxidant activity of NCB extracts by locality.

Locality	Mean %
Kamienka	21.26 ^d^
Zbojné	25.25 ^d,c^
Hôrka	25.62 ^d,c^
Miľpoš	26.30 ^c^
Lačnov	31.30 ^b^
Gallic acid	41.46 ^a^

Note: The differences between the localities according to ANOVA statistical analysis are marked with different letters.

**Table 4:** Multiple range analysis for antioxidant activity of CB extracts by locality.

Locality	Mean %
Gallic acid	43.60 ^c^
Hôrka	75.74 ^b^
Zbojné	77.12 ^a,b^
Miľpoš	79.15 ^a,b^
Lačnov	81.85 ^a^
Kamienka	82.82 ^a^

Note: The differences between the localities according to ANOVA statistical analysis are marked with different letters.
